# Effects of increasing tidal volume and end-expiratory lung volume on induced bronchoconstriction in healthy humans

**DOI:** 10.1186/s12931-024-02909-9

**Published:** 2024-08-07

**Authors:** Alessandro Gobbi, Andrea Antonelli, Raffaele Dellaca, Giulia M. Pellegrino, Riccardo Pellegrino, Jeffrey J. Fredberg, Julian Solway, Vito Brusasco

**Affiliations:** 1 Dipartimento di Elettronica, Informazione e Bioingegneria, Politecnico di Milano, Milano, 20133 Italy; 2 Restech Srl, Milano, 20124 Italy; 3 Allergologia e Fisiopatologia Respiratoria, ASO S. Croce e Carle, 12100 Cuneo, Italy; 4 Casa di Cura del Policlinico, Dipartimento di Scienze Neuroriabilitative, Milano, Italy; 5 Centro Medico Pneumologico Torino, 10129 Torino, Italy; 6 Harvard T. H. Chan School of Public Health, Harvard University, Boston, MA 02115 USA; 7 Department of Medicine, University of Chicago, Chicago, IL USA; 8 Dipartimento di Medicina Sperimentale, Università di Genova, 16132 Genova, Italy

**Keywords:** Lung volume, Tidal breathing, Airway caliber, Oscillometry, Methacholine

## Abstract

**Background:**

Increasing functional residual capacity (FRC) or tidal volume (V_T_) reduces airway resistance and attenuates the response to bronchoconstrictor stimuli in animals and humans. What is unknown is which one of the above mechanisms is more effective in modulating airway caliber and whether their combination yields additive or synergistic effects. To address this question, we investigated the effects of increased FRC and increased V_T_ in attenuating the bronchoconstriction induced by inhaled methacholine (MCh) in healthy humans.

**Methods:**

Nineteen healthy volunteers were challenged with a single-dose of MCh and forced oscillation was used to measure inspiratory resistance at 5 and 19 Hz (R_5_ and R_19_), their difference (R_5-19_), and reactance at 5 Hz (X_5_) during spontaneous breathing and during imposed breathing patterns with increased FRC, or V_T_, or both. Importantly, in our experimental design we held the product of V_T_ and breathing frequency (BF), *i.e*, minute ventilation (V_E_) fixed so as to better isolate the effects of changes in V_T_ alone.

**Results:**

Tripling V_T_ from baseline FRC significantly attenuated the effects of MCh on R_5_, R_19_, R_5-19_ and X_5_. Doubling V_T_ while halving BF had insignificant effects. Increasing FRC by either one or two V_T_ significantly attenuated the effects of MCh on R_5,_ R_19_, R_5-19_ and X_5_. Increasing both V_T_ and FRC had additive effects on R_5_, R_19_, R_5-19_ and X_5_, but the effect of increasing FRC was more consistent than increasing V_T_ thus suggesting larger bronchodilation. When compared at iso-volume, there were no differences among breathing patterns with the exception of when V_T_ was three times larger than during spontaneous breathing.

**Conclusions:**

These data show that increasing FRC and V_T_ can attenuate induced bronchoconstriction in healthy humans by additive effects that are mainly related to an increase of mean operational lung volume. We suggest that static stretching as with increasing FRC is more effective than tidal stretching at constant V_E_, possibly through a combination of effects on airway geometry and airway smooth muscle dynamics.

**Supplementary Information:**

The online version contains supplementary material available at 10.1186/s12931-024-02909-9.

## Introduction

Studies in animals and humans have brought clear evidence that increasing the operating lung volume, i.e., the end-expiratory lung volume above normal functional residual capacity (FRC) or the tidal volume (V_T_), reduces airway resistance [1, 2] and can attenuate [3] or reverse [4] the response to bronchoconstrictor stimuli. These effects of breathing at increased lung volume can be explained by either static or dynamic mechanisms. Since airways and lung parenchyma are interdependent, a static increase of lung volume is associated with an increase of airway caliber by the action of tethering forces opposing both the passive elastic recoil of the airway wall and the active contractile forces of airway smooth muscle. On the other hand, studies in-vitro have shown that dynamic swings can blunt the response of airway smooth muscle to contractile stimuli by mechanisms that reduce its force generation capacity [5, 6], though in bronchial segments this effect was observed only when pressure oscillations were raised to twice of those corresponding to normal V_T_ [7]. In vivo, increasing V_T_ [4], or breathing frequency (BF), or both [8] have a bronchodilator effect.

Therefore, it can be expected that increasing FRC or V_T,_ or their combinations, have beneficial effects in counteracting bronchoconstriction in vivo. However, in porcine bronchial segments, static hyper-distension reduced the maximal response to acetylcholine but blunted the relaxant effect of superimposed pressure oscillations of amplitude corresponding to twice the baseline V_T_ [9], raising the possibility that lung hyperinflation may compete with the bronchodilator effects of increasing V_T_ in vivo. In humans, the relative efficacy of physiologically relevant static hyperinflation and increased dynamic swings in countering airway narrowing has not been studied, but it can be hypothesized that they differ, owing to different underlying mechanisms.

To test this hypothesis, we designed the present study to evaluate whether the bronchodilator effect of breathing at increased lung volumes differs depending on whether attained by increasing FRC or V_T_. Moreover, we investigated whether the bronchodilator effects of increasing FRC and V_T_ were additive.

## Methods

### Subjects

Nineteen healthy volunteers (13 males/6 females) with no history respiratory/cardiovascular diseases participated in the study. No one was obese. Main anthropometric and respiratory functional data are reported in Table 1. Data were collected at Santa Croce and Carle Hospital (Cuneo, Italy), the protocol was approved by the local Ethical Committee, and each subject gave a written informed consent before participation.

**Table 1:** Subjects’ anthropometric characteristics and baseline lung functional data

Sex, M/F	13/6
Age, yr	37±9
Height, cm	174±9
BMI, kg·m^-2^	23±3
FEV_1_, L	4.27±0.90
FEV_1_, % of predicted	114±8
FEV_1_/VC	0.82±0.05
R_5_, cmH_2_O·s·L^-1^	2.08±0.44
R_19_, cmH_2_O·s·L^-1^	2.27±0.53
R_5-19_, cmH_2_O·s·L^-1^	-0.19±0.14
X_5_, cmH_2_O·s·L^-1^	-0.59±0.20

*BMI* Body mass index, *FEV*_*1*_ Forced expiratory volume in 1 s, *VC* slow inspiratory vital capacity, *R*_*5*_ Respiratory resistance at 5 Hz, *R*_*19*_ Respiratory resistance at 19 Hz, *R*_*5-19*_ difference in respiratory resistance between 5 and 19 *Hz; X*_*5*_ respiratory reactance at 5 Hz. Data are mean ± SD

### Measurements

Spirometry was measured by a mass flowmeter (SensorMedics Inc., CA, USA) following the ATS/ERS recommendations [10]. Respiratory impedance was measured by a forced oscillation technique (FOT) as previously described [11, 12]. Briefly, sinusoidal pressure oscillations (5 and 19 Hz; ~ 2 cmH_2_O peak-to-peak) were generated by a 16-cm diameter loudspeaker (model CW161N, Ciare, Italy) mounted in a rigid plastic box and connected in parallel to a mesh pneumotachograph and mouthpiece on one side and to a low-resistance high-inertance tube on the other side. Pressure oscillations were applied at the mouth during tidal breathing, while subjects had their cheeks supported by the hands of an investigator to minimize upper airway shunting. The overall load over the tidal breathing frequency range was 0.98 cm H_2_O•L^-1^•s. Airway opening pressure and flow were recorded by piezoresistive transducers (DCXL10DS and DCXL01DS Sensortechnics, Germany, respectively) and sampled at 200 Hz. A 15-L/min bias flow of air generated by an air pump (CMP08, 3A Health Care, Italy) was used to reduce dead space to about 35 ml. Pressure and flow signals were processed by a least-square algorithm [13, 14] to calculate respiratory resistance at 5 and 19 Hz (R_5_ and R_19_, respectively) and reactance at 5 Hz (X_5_). Artifacts due to glottis closure or expiratory airflow limitation were avoided by discarding breaths showing any of the following features: i) tidal volume <0.1 L or >2.0 L, ii) difference between measured flow oscillation and ideal sine wave with the same Fourier coefficients >0.2 [15], and iii) ratio of minimum to average X>3.5 [11]. The same breaths were used to measure V_T_, breathing frequency (BF), inspiratory and total time of each breath (T_I_ and T_Tot_, respectively), and estimate inspiratory drive (V_T_/T_I_), inspiratory duty cycle (T_I_/T_Tot_), and minute ventilation (V_E_).

### Protocol

#### Pre-study day

Subjects attended the laboratory for spirometry and determination of the dose of methacholine (MCh) to be used for the study day. For this purpose, after baseline FOT measurements, MCh chloride dry-powder (Laboratorio Farmaceutico Lofarma, Milan, Italy) was dissolved in distilled water and administered by an ampoule-dosimeter system (MB3 MEFAR, Brescia, Italy) delivering aerosol particles with a median mass diameter of 1.53-1.61μm, while subjects breathed quietly in a sitting position. The starting dose was of 300 μg followed by doubling doses until R_5_ increased by at least 100% from baseline.

#### Study day

**Fig. 1:**
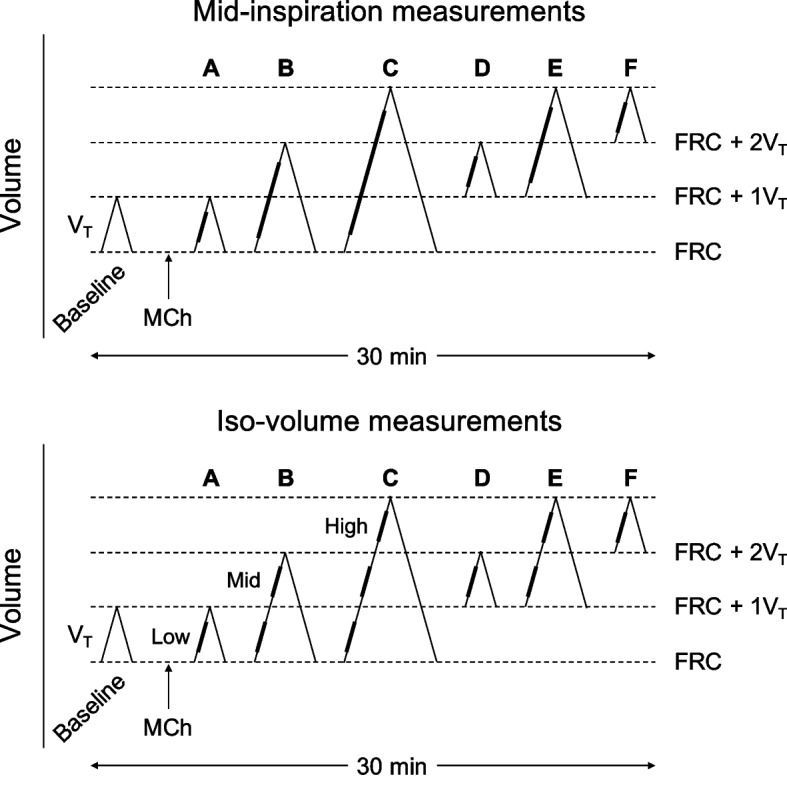
Patterns of breathing before after methacholine (MCh) with tidal volume (V_T_) initiated from spontaneous or increased functional residual capacity (FRC). For each condition, respiratory impedance measures were calculated over the 3 mid-quintiles of the whole inspiratory phase (upper panel) or over the 3 mid-quintiles of iso-volume inspiratory portions (lower panel) as shown by the thick lines

Baseline FOT measurements were taken during 2 min of spontaneous tidal breathing. Then, the subjects were trained to breathe, by using visual feed-back of spirometry tracing, for 2 min with imposed combinations of FRC or V_T_. Thereafter, each subject inhaled a single dose of MCh equal to the last dose given on the pre-study day and R_5_ was measured 2 min later during spontaneous tidal breathing to confirm the persistence of bronchoconstriction. Then, FOT measurements were taken while subjects maintained for 2 min each of the following imposed breathing patterns in randomized order (Fig. 1): A) spontaneous V_T_ from spontaneous FRC, B) near double V_T_ from spontaneous FRC, C) near triple V_T_ from spontaneous FRC, D) spontaneous V_T_ from FRC increased by 1 V_T_, E) near double V_T_ from FRC increased by 1 V_T_, and F) spontaneous V_T_ from FRC increased by 2 V_T_. For each V_T_ increase the subjects were asked to adjust BF to prevent large increments of V_E_. Before each change of breathing pattern, R_5_ was measured during spontaneous tidal breathing to check for the stability of bronchoconstriction. If R_5_ was 10% or more lower than initial post-MCh value an additional half dose of MCh was given to restore bronchoconstriction. This happened occasionally in 6 subjects, with no relation to any specific breathing pattern. At the end of the study, aerosol albuterol was administered to relieve symptoms if any.

### Data analysis

For each breathing pattern, R_5_, R_19_, R_5-19_, and X_5_ were calculated over the 3 mid-quintiles of the whole inspiratory phase (Fig. 1, upper panel) or over the 3 mid-quintiles of iso-volume inspiratory portions (Fig. 1, lower panel).

Differences in R_5_, R_19_, R_5-19_, X_5_, V_T_, BF, V_T_/T_I_, T_I_/T_Tot_, and V_E_ between conditions were tested for statistical significance by a one-way repeated-measure analysis of variance (ANOVA) with Holm-Sidak post-hoc test for multiple-comparisons. Values of p<0.05 were considered statistically significant. Data are presented as mean ± standard deviation (SD).

## Results

### Breathing patterns during the experimental conditions

The spontaneous breathing pattern after MCh (A) did not differ significantly from the spontaneous pattern before methacholine (Table 2). V_T_ and BF changed with the imposed *patterns (B-F)* as per protocol. Even though great attention was paid to maintain V_E_ as constant as possible among the imposed breathing patterns, it was with *patterns C, E, and F* that V_E_ slightly but significantly increased than with patterns than A and B. These differences were associated with significant differences in mean inspiratory, V_T_/T_I_. Neither V_E_ nor V_T_/T_I_ were significantly different among breathing *patters C, D, E, and F*. There were no significant differences in T_I_/T_TOT_ among all breathing patterns.

**Table 2:** Patterns of breathing during experimental conditions

Breathing pattern	V_T_, L	BF, min^-1^	V_E_, L·min^-1^	V_T_/T_I_, L·s^-1^	T_I_/ T_Tot_
Baseline					
Spontaneous	0.85±0.29	12±3	9.9±2.6	0.40±0.10	0.41±0.06
After methacholine					
FRC, V_T_ (A)	0.84±0.29	12±3	9.5±2.5	0.37±0.09	0.43±0.05
FRC, 2V_T_ (B)	1.49±0.61^♦^	7±2^◊^	10.1±3.2	0.41±0.16	0.43±0.08
FRC, 3V_T_ (C)	2.07±0.49^†^	6±1^╫^	12.8±3.7^■^	0.51±0.22^■^	0.44±0.08
FRC_+1VT_, V_T_ (D)	0.96±0.43^*^	13±3^‡^	11.2±3.1	0.48±0.21	0.41±0.08
FRC_+1VT_, 2V_T_ (E)	1.45±0.35^♦^	9±2^◊^	12.8±4.9^■^	0.55±0.25^◊^	0.40±0.08
FRC_+2VT_, V_T_ (F)	0.89±0.28^*^	14±3^#^	12.9±5.1^■^	0.51±0.23^■^	0.43±0.06
ANOVA	*p*<0.001	*p*<0.001	*p*<0.001	*p*<0.001	*p*=0.26
	^♦^*p*<0.001 vs. A, C, D, F.	^‡^*p*<0.001 vs. B, C, E.	^□^*p*=0.03 vs. C, E, F.	^■^*p*=0.02 vs. A	
	^*^p<0.001 vs. B, C, E.	^◊^*p*<0.001 vs. A, C, D, F.	^■^*p*=0.004 vs. A;	^◊^*p*<0.001 vs. A	
	^†^*p*<0.001 vs. A, B, D, E, F.	^╫^*p*<0.001 vs. A, D, E, F.	*p*=0.03 vs. B.	* p*=0.02 vs. B	
		^#^*p*<0.001 vs. A, B, C, E.			

*V*_*T*_ tidal volume, *BF* Breathing frequency, *V*_*E*_, minute ventilation, *V*_*T*_*/T*_*i*_ mean inspiratory flow, *T*_*i*_*/ T*_*tot*_ respiratory duty cycle; FRC, functional residual capacity. Data are mean ± SD

### Mid-inspiration measures

**Fig. 2:**
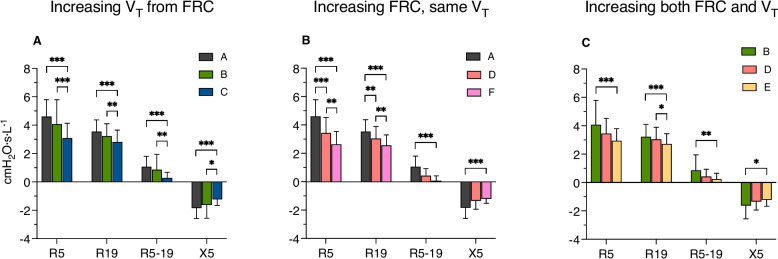
Effects of increasing tidal volume from spontaneous functional residual capacity (patterns **A**, **B**, **C**) (**A**), increasing functional residual capacity with spontaneous (patterns **A**, **D**, **F**) (**B**), or both (patterns **B**, **E**) (**C**) on mid-inspiration impedance measures. Effects of patterns achieving the same peak volume (**C** vs. **E** and vs. **F**) on mean-inspiratory impedance measurements (**D**). V_T_, tidal volume; FRC, functional residual capacity. R_5_, respiratory resistance at 5 Hz, R_19_, respiratory resistance at 19 Hz; R_5-19_, difference in respiratory resistance between 5 and 19 Hz; X_5_, respiratory reactance at 5 Hz. Columns heights indicate means and error bars standard deviations. *, *p*<0.005; **, *p*<0.01; *p*<0.001

In general, breathing at increased FRC, increased V_T_, or both attenuated the changes induced by MCh inhalation on R_5_, R_19_, R_5-19_, and X_5_ (Fig. 2 and Supplemental Table 1).

Increasing V_T_ from spontaneous FRC was associated with significant reductions of R_5_, R_19_, R_5-19_ and less negative X_5_ when V_T_ was tripled (*pattern C*) but not doubled (*pattern B*) compared to spontaneous breathing (*pattern A*) V_T_. Yet, the attenuating effects of *pattern C* were significantly greater than those of *pattern B*.

Increasing FRC by either one (*pattern D*) or two (*pattern F*) V_T_ with constant spontaneous V_T_ was associated with significant reductions of R_5_ and R_19_ than *pattern A,* while R_5-19_ was significantly reduced and X_5_ less negative with *pattern F* but not *pattern D*.

Increasing both V_T_ and FRC (*pattern E*) was associated with significantly lower R_5_, R_19_, R_5-19_ and less negative X_5_ than increasing V_T_ alone (*pattern B*) and significantly lower R_19_ than increasing FRC alone (*pattern D*).

Breathing patterns with the same peak volume, no matter whether achieved by increasing V_T_ or FRC or both (*patterns B* vs.* D and C* vs.* E* and vs. *F*) showed insignificantly different effects on airway narrowing.

Notably, R_5_ (cmH_2_O•L^-1^•s) was reduced by 0.57±1.18 when V_T_ was doubled (*pattern B* vs *pattern A),* by 1.19±0.70 when FRC was increased by 1 V_T_ (*pattern D* vs *pattern A*), and by 1.84±0.88 when both V_T_ and FRC were increased (*pattern E* vs *pattern A*). Similarly, R_19_ (cmH_2_O•L^-1^•s) was reduced by 0.29±0.35 when V_T_ was doubled (*pattern B* vs *pattern A)*, by 0.48±0.46 when FRC was increased by 1 V_T_ (*pattern D* vs *pattern A*), and by 0.91±0.42 when both V_T_ and FRC were increased (*pattern E* vs *pattern A*). These results suggest simply additive effects, but the increase of FRC was more potent to mitigate airway narrowing than the increase in V_T_.

### Iso-volume measures

**Fig. 3:**
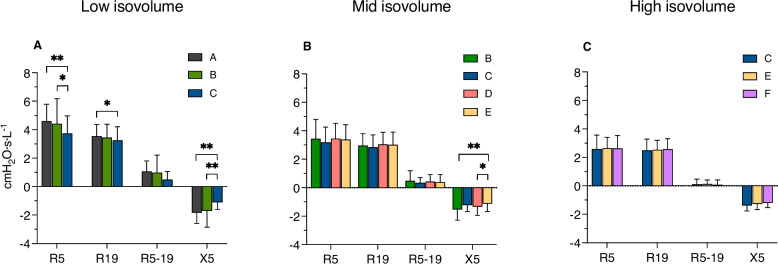
Effects of increasing tidal volume from spontaneous (patterns **A**, **B**, **C**) or increased (patterns **D**, **E**, **F**) functional residual capacity on iso-volume inspiratory impedance measures. Other abbreviations as in Fig. 2. Columns heights indicate means and error bars standard deviations. *, *p*<0.005; **, *p*<0.01

In general, R_5_, R_19_, and R_5-19_ were inversely related to the lung volume at which they were measured (Fig. 3 and Supplemental Table 2), while the X_5_ values were inconsistently related to lung volumes.

At low iso-volume, R_5_ and R_19_, were significantly lower and X_5_ was less negative than during spontaneous breathing (*pattern A*) when V_T_ was tripled (*pattern C*) but not doubled (*pattern B*). Yet, the attenuating effects of *pattern C* on R_5_ and X_5_ were significantly greater than those of *pattern B*.

At mid iso-volume, R_5_, R_19_, and R_5-19_ did not differ significantly with increments of V_T_ (*patterns B and C)*, or FRC (*pattern D*), or both (*pattern E*). However, X_5_ was significantly less negative when both FRC and V_T_ were increased (*pattern E*) than when V_T_ (*pattern B*) or FRC (*pattern D*) were increased alone.

At high iso-volume, there were no significant differences with increments of V_T_ (*pattern C*), or FRC (*panel F*), or both (*panel E*).

## Discussion

The main findings of the present study in healthy volunteers were that 1) the changes of respiratory impedance induced by inhaled MCh were significantly attenuated by increasing FRC, or V_T_, or both, 2) increasing FRC had more consistent effects than increasing V_T_, 3) the effects of increasing FRC and V_T_ were additive, and ) volume-independent effects attributable to tidal stretching were observed only when V_T_ was three times larger than during spontaneous breathing.

### Comments on methodology

We used oscillometry because it is the only available method enabling intra-breath measurements of respiratory mechanics over specific portions of lung volume during tidal breathing, but it has two major limitations. First, oscillometry does not directly measure airway resistance but also lung tissue and chest wall resistances. Airway resistance is inversely related to V_T_ whereas lung tissue resistance is inversely related to BF [2]. Therefore, it is possible that the effects of increasing V_T_ on airway caliber were counteracted by the effects of decreasing BF on tissue resistance. We think this had no major effect on our results because the attenuation of R_5_, which reflects in large part tissue resistance, was not less than the attenuation of R_19_, which mainly reflect airway resistance. Second, breathing at increased lung volumes requires activation of inspiratory muscles, which increases chest wall elastance [16]. Therefore, we cannot exclude that changes in X_5_ with different breathing patterns were counteracted by changes in chest wall stiffness.

Although our subjects were asked to maintain V_E_ as constant as possible by decreasing BF when V_T_ was increased, there was a tendency for V_E_ to increase (Table 2), thus likely resulting in an increased alveolar ventilation and airway hypocapnia, mainly when achieved by increasing V_T_. Hypocapnia has a bronchoconstrictor effect [17], thus possibly counteracting the bronchodilator effects of imposed breathing patterns. We did not measure end-tidal CO_2_, but we believe this had no major impact on our results for two reasons. First, assuming normal anatomical plus instrumental dead space and CO_2_ production, we estimated a mean difference in alveolar PCO_2_ between patterns C and A to be approximately 7 mmHg, which was reported to have insignificant effects on the respiratory impedance of healthy subjects [18]. Second, the differences in V_E_ between any imposed patters were insignificant and differences in alveolar PCO_2_ presumably minimal.

Finally, for changes in V_T_ were associated with changes in BF and the ratio T_I_/T_TOT_ remained constant, the effects of tissue viscoelasticity could not be evaluated. Nevertheless, breathing patterns with low BF would have increased the time for airway smooth muscle relaxation during the inspiratory phase but also for re-shortening during the expiratory phase.

### Interpretation of results

The present study was designed on the premises that both lung hyperinflation and increased breathing depth are mechanisms protecting against airway narrowing, but their relative efficacies are unknown.

That increasing lung volume is associated with a proportional increase of airway conductance, i.e., the reciprocal of airway resistance, was first reported in 1958 by Briscoe and Dubois [1] and subsequently confirmed in excised animal [19] and human [20] lungs with relaxed airways. This effect was simply attributed to a geometric change of airways being distended by the static radial traction of the surrounding lung parenchyma. Studies in contracted airway smooth muscle strips have consistently shown that sustained step-changes of length can rapidly attenuate active tension, possibly due to disassembly of the contractile apparatus, followed by a gradual recovery due to length adaptation [20, 21]. By contrast, in whole bronchial segments a sustained inflationary increase of transmural pressure also caused an immediate reduction in tension, but this was followed by a continuous gradual decrease [22]. Airway wall stiffening was proposed to explain the difference between intact bronchi and muscle strips [22, 23]. In our study, R_5_ was stable or decreased between the different breathing patterns, but never increased, which makes the occurrence of length adaptation unlikely. Thus, it is possible that the attenuations of airway narrowing we observed after 2 min of breathing at increased FRC reflected not only geometric changes in airway caliber but also mechanisms opposing both the passive elastic recoil of the airway wall and the active contractile forces of airway smooth muscle.

The inhibitory effect of cycling stretching on airway smooth muscle active force generation has been reported consistently in both isolated muscle strips [5, 6] and isolated bronchial segments [7]. It is well-established in animals [7] and humans [4, 24] that the magnitude of the bronchodilator effects of tidal breathing increases with increasing frequency of breathing and with increasing tidal volume. Two independent lines of evidence suggest, further, that the attenuation of smooth muscle contractile force is attributable to changes of V_E_, which is the product V_T_ x BF, independently of changes of either V_T_ or BF taken individually [24, 25]. Equivalently, neither the amplitude of tissue cyclic strain nor the cyclic frequency is as important as their product, namely, the amplitude of the tissue strain rate. To assess this phenomenon still further, in this report we used an experimental design in which we held the product V_T_ x BF fixed so as to better isolate the effects of changes in V_T_ alone. This is an important issue in our study, as we see that when V_E_ could not be kept constant (*pattern C vs A*) the impedance values at low iso-volume were significantly attenuated presumably because of the higher mean inspiratory flow (V_T_/T_I_ ) causing a faster lung stretching rate rather than the increase in V_T_ itself.

Three theories can be invoked to explain the above findings [26], namely, that stretching of airway smooth muscle causes a plastic rearrangement of the contractile apparatus [6, 27, 28], or modifies the crossbridge cycling rate and latch bridges formation [5] or causes temporary detachment of attached cross bridges [29].

In an attempt to examine the relative bronchodilator effects of static hyperinflation and dynamic stretching, we measured inspiratory impedance in healthy subjects with MCh-induced bronchoconstriction breathing with different combinations of FRC and V_T_. As expected, increasing either V_T_ or FRC significantly attenuated the changes induced by MCh on R_5_ and R_19_, R_5-19_, suggestive of a generalized increase of airway caliber, but also decreased R_5-19_ and made X_5_ less negative. To the extent that an increase in R_5-19_ and a decrease in X_5_ reflect heterogeneous distribution of time constants within the lung periphery [30], the significant improvement of these variables with the increase in FRC and V_T_ (Figs. 2 and 3) suggests that increasing lung volumes no matter how it was achieved made ventilation more homogeneous. While the effects of increasing V_T_ on R_5_ and R_19_ were significant only when it was threefold the spontaneous V_T_, the effects of increasing FRC where already significant when it was increased by one V_T_, suggesting a more consistent effect of increasing static than dynamic tidal stretching.

The effects of increasing both V_T_ and FRC were additive, *i.e.*, the effect of dynamic stretching was not blunted by an increased static stretch. This finding is in apparent contradiction with a study showing that in isolated bronchial segments hyperinflation blunted the effect of pressure oscillations corresponding to twice a normal V_T_ [9] In that study, bronchi were hyperinflated at a transmural pressure of 20 cmH_2_O, where airway compliance is reduced [7] and so are the amplitude of volume oscillation and airway smooth muscle strain. Examining our data in the light of a previous study [31], (Fig. 3), we estimate that the largest end-tidal inspiratory volumes achieved as with *patterns C, E and F* would have not exceed the values associated with transpulmonary pressures in excess of 20 cm H_2_O. Since bronchial transmural pressure might differ from transpulmonary pressure in the presence of bronchoconstriction [32], we cannot exclude that stress on airway walls increased with the increase of end-inspiratory volume. Therefore, the increments of V_T_ in our study were likely to reflect increments of airway smooth muscle strain but not stress. The latter, however, does not seem to be the major determinant of the decrease in airway smooth muscle contractility with breathing maneuvers [33, 34].

The fact that the effects of FRC and V_T_ were simply additive does suggest that lung hyperinflation and tidal swings operated via a similar mechanism, *viz.* increase of operational lung volume. This interpretation is supported by the lack of differences at iso-volumes among most breathing patterns. The only exceptions were the lower R_5_, R_19_, R_5-19_, and less negative X_5_ at low lung volume after triple V_T_ and the less negative X_5_ at mid lung volume with breathing patterns with the highest end-inspiratory lung volume, *i.e.*, tripling V_T_ (*pattern C*) and doubling V_T_ from increased FRC (*pattern E*). These findings are consistent with a study in airway segments showing modest dilator effects with peak-to-peak pressure oscillations of 10 but not 5 cmH_2_O [7]. As FOT measurement were taken during the inspiratory phase, these findings possibly reflect volume-independent dynamic effects on airway smooth muscle persisting after the expiratory phase, even when BF and, in turn, expiratory time for re-narrowing was the largest (*pattern C*).

Why was hyperinflation more potent than tidal swings against airway narrowing in the present study is a matter of speculation. Increasing either FRC or V_T_ results in increased mean operational lung volume, which is associates with an increase of airway caliber owing to the tethering force of lung parenchyma opposing the passive elastic recoil of airway walls. However, the mechanisms of static and dynamic stretching on airway smooth muscle active force may be different. One possibility is that in our study the sustained increments of operational lung volume maintained the airway smooth muscle in a condition of reduced force generation capacity by disassembling the contractile apparatus before the occurrence of length adaptation [20, 21] or substantial reduction of tethering force due to stress relation of lung parenchyma [35]. By contrast, additional time-dependent effects of tidal stretching, *e.g.*, on cross-bridge cycling rate, were possibly obscured by the re-constriction during expiratory phase unless started from very high end-inspiratory volume. Another possible mechanism explaining the larger bronchodilator effects yielded by the increase in FRC rather than V_T_ could be the larger amount of nitric oxide penetrating the airway lumen when narrowing is relieved by distending lung parenchyma [36].

The results of the present study in healthy subjects cannot be directly extrapolated to asthma because the mechanisms regulating airway smooth muscle contractility and heterogeneity of ventilation may differ in disease. Yet, it is known that FRC increases in asthma with the occurrence of expiratory flow limitation [37] and decreases after bronchodilator treatments [38]. Moreover, some beneficial effects of continuous positive airway pressure against airway responsiveness have been reported. To what extent hyperinflation can alleviate asthma symptoms remains to be elucidated, considering that above a given threshold it may cause an increase of inspiratory work of breathing [39] and limit the increase in V_T_ [21].

In conclusion, this study provides evidence that both lung hyperinflation and increased tidal stretching yield substantial bronchodilatation in human lungs exposed to a constrictor agent, though the former seems more effective than the latter presumably because of additive effects on airway smooth muscle contractile force and non-contractile airway tissues.

### Supplementary Information

Supplementary Material 1.

## Data Availability

The data that support the findings of this study are available from the authors and are available upon request.

## References

[1] Briscoe WA. The relationship between airway resistance, airway conductance and lung volume in subjects of different age and body size. J Clin Invest. 1958;37:1279–85. 10.1172/JCI103715. 13575526 10.1172/JCI103715 PMC1062796

[2] Brusasco V, Warner DO, Beck KC, Rodarte JR, Rehder K. Partitioning of pulmonary resistance in dogs: effect of tidal volume and frequency. J Appl Physiol. 1989;66:1190–6. 10.1152/jappl.1989.66.3.1190. 2708244 10.1152/jappl.1989.66.3.1190

[3] Ding DJ, Martin JG, Macklem PT. Effects of lung volume on maximal methacholine-induced bronchoconstriction in normal humans. J Appl Physiol. 1987;62:1324–30. 3553143 10.1152/jappl.1987.62.3.1324

[4] Salerno FG, Pellegrino R, Torchio G, Spanevello A, Brusasco V, Crimi E. Attenuation of induced bronchoconstriction in healthy subjects: effects of breathing depth. J Appl Physiol. 2005;98:817–21. 10.1152/japplphysiol.00763.2004. 15475599 10.1152/japplphysiol.00763.2004

[5] Oliver MN, Fabry B, Marinkovic A, Mijailovich SM, Butler JP. Fredberg JJ Airway hyperresponsiveness, remodeling, and smooth muscle mass: right answer, wrong reason? Am J Respir Cell Mol Biol. 2007;37:264–72. 17463392 10.1165/rcmb.2006-0418OC PMC1994228

[6] Gunst SJ, Meiss RA, Wu MF, Rowe M. Mechanisms for the mechanical plasticity of tracheal smooth muscle. Am J Physiol. 1995;268:C1267–76. 7762621 10.1152/ajpcell.1995.268.5.C1267

[7] LaPrad AS, Szabo TL, Suki B, Lutchen KR. Tidal stretches do not modulate responsiveness of intact airways in vitro. J Appl Physiol. 2010;109:295–304. 10.1152/japplphysiol.00107.2010. Epub 2010 Apr 29. PMID: 20431023; PMCID: PMC2928594. 20431023 10.1152/japplphysiol.00107.2010 PMC2928594

[8] Shen X, Gunst SJ, Tepper RS. Effect of tidal volume and frequency on airway responsiveness in mechanically ventilated rabbits. J Appl Physiol. 1997;83:1202–8. 10.1152/jappl.1997.83.4.1202. 9338429 10.1152/jappl.1997.83.4.1202

[9] Cairncross A, Noble PB, McFawn PK. Hyperinflation of bronchi in vitro impairs bronchodilation to simulated breathing and increases sensitivity to contractile activation. Respirology. 2018;23:750–5. 10.1111/resp.13271. Epub 2018 Feb 20 PMID: 29462842. 29462842 10.1111/resp.13271

[10] Miller M, Hankinson J, Brusasco V, Burgos F, Casaburi R, Coates A, Crapo R, Enright P, van der Grinten CPM, Gustafsson P, Jensen R, Johnson DC, MacIntyre N, McKay R, Navajas D, Pedersen OF, Pellegrino R, Viegi G, Wanger J. Standardization of spirometry. Eur Respir J. 2005;26:319–38. 16055882 10.1183/09031936.05.00034805

[11] Dellacà RL, Gobbi A, Pastena M, Pedotti A, Celli B. Home monitoring of within-breath respiratory mechanics by a simple and automatic forced oscillation technique device. Physiol Meas. 2010;31(4):N11. 20182000 10.1088/0967-3334/31/4/N01

[12] Gobbi A, Milesi I, Govoni L, Pedotti A, Dellaca RL. A new telemedicine system for the home monitoring of lung function in patients with obstructive respiratory diseases. eHealth, Telemedicine, and Social Medicine, 2009. eTELEMED'09. International Conference (pp. 117-122). IEEE.

[13] Kaczka DW, Barnas GM, Suki B, Lutchen KR. Assessment of time-domain analyses for estimation of low-frequency respiratory mechanical properties and impedance spectra. Ann Biomed Eng. 1995;23:135–51. 7605051 10.1007/BF02368321

[14] Kaczka DW, Ingenito EP, Lutchen KR. Technique to determine inspiratory impedance during mechanical ventilation: implication for flow limited patients. Ann Biomed Eng. 1999;27:340–55. 10374726 10.1114/1.146

[15] Marchal F, Schweitzer C, Demoulin B, Chone C, Peslin R. Filtering artefacts in measurements of forced oscillation respiratory impedance in young children. Physiol Meas. 2004;25:1153–66. 15535181 10.1088/0967-3334/25/5/006

[16] Barnas GM, Heglund NC, Yager D, Yoshino K, Loring SH, Mead J. Impedance of the chest wall during sustained respiratory muscle contraction. J Appl Physiol. 1989;66:360–9. 10.1152/jappl.1989.66.1.360. PMID: 2917942. 2917942 10.1152/jappl.1989.66.1.360

[17] Newhouse MT, Becklake MR, Macklem PT, McGregor M. Effect of alterations in end-tidal CO_2_ tension on flow resistance. J Appl Physiol. 1964;19:745–9. 14195587 10.1152/jappl.1964.19.4.745

[18] van den Elshout FJ, van Herwaarden CL, Folgering HT. Effects of hypercapnia and hypocapnia on respiratory resistance in normal and asthmatic subjects. Thorax. 1991;46:28–32. 10.1136/thx.46.1.28. PMID: 1908137; PMCID: PMC1020910. 1908137 10.1136/thx.46.1.28 PMC1020910

[19] Hughes JMB, Hoppin FG, Mead J. Effect of lung inflation on bronchial length and diameter in excised lungs. J Appl Physiol. 1972;32:25–35. 5007013 10.1152/jappl.1972.32.1.25

[20] Wilson AG, Massarella GR, Pride NB. Elastic properties of airways in human lungs post mortem. Am Rev Respir Dis. 1974;110:716–29. 4429267 10.1164/arrd.1974.110.6P1.716

[21] Bossé Y, Sobieszek A, Paré PD, Seow CY. Length adaptation of airway smooth muscle. Proc Am Thorac Soc. 2008;5:62–7. 18094086 10.1513/pats.200705-056VS

[22] Bossé Y. The Strain on Airway Smooth Muscle During a Deep Inspiration to Total Lung Capacity. J Eng Sci Med Diagn Ther. 2019;2:0108021–01080221. 10.1115/1.4042309. 32328568 10.1115/1.4042309 PMC7164505

[23] Ansell TK, McFawn PK, McLaughlin RA, Sampson DD, Eastwood PR, Hillman DR, Mitchell HW, Noble PB. Does smooth muscle in an intact airway undergo length adaptation during a sustained change in transmural pressure? J Appl Physiol. 2015;118:533–43. 10.1152/japplphysiol.00724.2014. 25729015 10.1152/japplphysiol.00724.2014

[24] Torchio R, Gobbi A, Gulotta C, Antonelli A, Dellacà R, Pellegrino GM, Pellegrino R, Brusasco V. Role of hyperpnea in the relaxant effect of inspired CO_2_ on methacholine-induced bronchoconstriction. J Appl Physiol. 2022;132:1137–44. 10.1152/japplphysiol.00763.2021. Epub 2022 Mar 31. 35358399 10.1152/japplphysiol.00763.2021

[25] Oliver M, Kováts T, Mijailovich SM, Butler JP, Fredberg JJ, Lenormand G. Remodeling of integrated contractile tissues and its dependence on strain-rate amplitude. Phys Rev Lett. 2010;105(15):158102. 10.1103/PhysRevLett.105.158102. Epub 2010 Oct 4. 21230941 10.1103/PhysRevLett.105.158102 PMC3940190

[26] Doeing DC, Solway J. Airway smooth muscle in the pathophysiology and treatment of asthma. J Appl Physiol. 2013;114:834–43. 23305987 10.1152/japplphysiol.00950.2012 PMC3633438

[27] Gunst S, Stropp JQ, Service J. Mechanical modulation of pressure-volume characteristics of contracted canine airways in vitro. J Appl Physiol. 1990;68:2223–9. 2361927 10.1152/jappl.1990.68.5.2223

[28] Pratusevich VR, Seow CY, Ford LE. Plasticity in canine airway smooth muscle. J Gen Physiol. 1995;105:73–94. 7730790 10.1085/jgp.105.1.73 PMC2216929

[29] Luo L, Wang L, Paré PD, Seow CY, Chitano P. The Huxley crossbridge model as the basic mechanism for airway smooth muscle contraction. Am J Physiol Lung Cell Mol Physiol. 2019;317:L235–46. 10.1152/ajplung.00051.2019. Epub 2019 May 22. PMID: 31116578; PMCID: PMC6734385. 31116578 10.1152/ajplung.00051.2019 PMC6734385

[30] LaPrad AS, Lutchen KR. Respiratory impedance measurements for assessment of lung mechanics: focus on asthma. Respir Physiol Neurobiol. 2008;163:64–73. 10.1016/j.resp.2008.04.015. 18579455 10.1016/j.resp.2008.04.015 PMC2637462

[31] Pellegrino R, Pompilio P, Quaranta M, Aliverti A, Kayser B, Miserocchi G, Fasano V, Cogo A, Milanese M, Cornara G, Brusasco V, Dellacà R. Airway responses to methacholine and exercise at high altitude in healthy lowlanders. J Appl Physiol. 2010;108:256–65. 19940099 10.1152/japplphysiol.00677.2009

[32] Winkler T. Airway Transmural Pressures in an Airway Tree During Bronchoconstriction in Asthma. J Eng Sci Med Diagn Ther. 2019;2:0110051–6. 10.1115/1.4042478. Epub 2019 Feb 13. PMID: 32328574; PMCID: PMC7164500. 32328574 10.1115/1.4042478 PMC7164500

[33] Gobbi A, Pellegrino R, Gulotta C, Antonelli A, Pompilio P, Crimi C, Torchio R, Dutto L, Parola P, Dellacà RL, Brusasco V. Short-term variability in respiratory impedance and effect of deep breath in asthmatic and healthy subjects with airway smooth muscle activation and unloading. J Appl Physiol. 2013;115:708–15. 10.1152/japplphysiol.00013.2013. Epub 2013 Jun 13 PMID: 23766502. 23766502 10.1152/japplphysiol.00013.2013

[34] Pascoe CD, Seow CY, Paré PD, Bossé Y. Decrease of airway smooth muscle contractility induced by simulated breathing maneuvers is not simply proportional to strain. J Appl Physiol. 2013;114:335–43. 23195632 10.1152/japplphysiol.00870.2012

[35] Rodarte JR, Noredin G, Miller C, Brusasco V, Pellegrino R. Lung elastic recoil during breathing at increased lung volume. J Appl Physiol. 1999;87:1491–5. 10.1152/jappl.1999.87.4.1491. 10517783 10.1152/jappl.1999.87.4.1491

[36] Karamaoun C, Haut B, Van Muylem A. A new role of the exhaled nitric oxide as a functional marker of peripheral airway caliber changes; a theoretical study. J Appl Physiol. 2018;124:1025–33. 29357478 10.1152/japplphysiol.00530.2017

[37] Pellegrino R, Violante B, Nava S, Rampulla C, Brusasco V, Rodarte JR. Expiratory airflow limitation and hyperinflation during methacholine-induced bronchoconstriction. J Appl Physiol. 1993;75:1720–7. 10.1152/jappl.1993.75.4.1720. 8282625 10.1152/jappl.1993.75.4.1720

[38] Woolcock AJ, Read J. Lung volumes in exacerbations of asthma. Am J Med. 1966;41:259–73. 10.1016/0002-9343(66)90021-0. 5912303 10.1016/0002-9343(66)90021-0

[39] Lougheed MD, Lam M, Forkert L, Webb KA, O’Donnell DE. Breathlessness during acute bronchoconstriction in asthma. Pathophysiologic mechanisms Am Rev Respir Dis. 1993;148:1452–9. 10.1164/ajrccm/148.6_Pt_1.1452. 8256884 10.1164/ajrccm/148.6_Pt_1.1452

