# Supplementary material for: Effects of increasing tidal volume and end-expiratory lung volume on induced bronchoconstriction in healthy humans
Source: Respir Res. 2024 Aug 7;25:298. doi: 10.1186/s12931-024-02909-9 (PMC11304934; doi:10.1186/s12931-024-02909-9)
Supplement: Supplementary file 1 — Supplementary Material 1. [file 12931_2024_2909_MOESM1_ESM.docx]

Supplemental Table 1. Respiratory impedance data measured over mid inspiratory phase.

| Breathing pattern | | R_5_, cm H_2_O•L^-1^•s | R_19_, cm H_2_O•L^-1^•s | R_5-19_, cm H_2_O•L^-1^•s | X_5_, cm H_2_O•L^-1^•s |
| --- | --- | --- | --- | --- | --- |
| Baseline | | | | | |
| Spontaneous | | 2.08±0.44 | 2.27±0.53 | -0.20±0.14 | -0.59±0.20 |
| After methacholine | | | | | |
| FRC, V_T_ (A) | | 4.59±1.20 | 3.53±0.84 | 1.06±0.75 | -1.82±0.76 |
| FRC, 2V_T_ (B) | | 4.05±1.73 ♦ | 3.20±0.90 ♦ | 0.85±1.10 ♦ | -1.61±0.95 ♦ |
| FRC, 3V_T_ (C) | | 3.07±1.06 † | 2.81±0.84 † | 0.26±0.41 † | -1.22±0.43 † |
| FRC_+1VT_, V_T_ (D) | | 3.43±1.09 § | 3.02±0.87 § | 0.41±0.52 § | -1.32±0.62 § |
| FRC_+1VT_, 2V_T_ (E) | | 2.93±0.86 # | 2.70±0.74 # | 0.23±0.43 # | -1.20±0.47 # |
| FRC_+2VT_, V_T_ (F) | | 2.62±0.92 ^╫^ | 2.56±0.75 ^╫^ | 0.06±0.35 ^╫^ | -1.18±0.34 ^╫^ |
| ANOVA | p <0.001  ♦ p<0.001 vs. C, E, F  † p<0.001 vs. A, B  § p<0.001 vs. A  p=0.003 vs. F  # p<0.001 vs. A, B  ^╫^ p<0.001 vs. A, B  p=0.003 vs. D | | p<0.001  ♦ p=0.005 vs. C  p<0.001 vs. E, F  † p<0.001 vs. A  p=0.005 vs. B  § p=0.002 vs. A  p=0.02 vs. E  p=0.001 vs. F  # p<0.001 vs. A, B  p=0.02 vs. D  ^╫^ p<0.001 vs. A, B  p=0.001 vs. D | p<0.001  ♦ p=0.004 vs. C  p=0.002 vs. E  p<0.001 vs. F  † p<0.001 vs. A  p=0.004 vs. B  § p=0.002 vs. A  # p<0.001 vs. A,  p=0.002 vs. B  ^╫^ p <0.001 vs. A, B | p<0.001  ♦ p=0.02 vs. C  p=0.01 vs. E  p=0.008 vs. F  † p<0.001 vs. A  p=0.02 vs. B  § p=0.003 vs. A  # p<0.001 vs. A  p=0.01 vs. B  ^╫^ p<0.001 vs. A  p=0.008 vs. B |

R_5_, respiratory resistance at 5 Hz, R_19_, respiratory resistance at 19 Hz; R_5-19_, difference in respiratory resistance between 5 and 19 Hz; X_5_, respiratory reactance at 5 Hz. V_T_, tidal volume; FRC, functional residual capacity. Data are mean ± SD.

Supplemental Table 2. Respiratory impedance data measured at iso-volumes.

| Breathing pattern | R_5_, cm H_2_O•L^-1^•s | R_19_, cm H_2_O•L^-1^•s | R_5-19_, cm H_2_O•L^-1^•s | | X_5_, cm H_2_O•L^-1^•s |
| --- | --- | --- | --- | --- | --- |
| Low iso-volume | | | | | |
| FRC, V_T_ (A) | 4.59±1.20 | 3.53±0.84 | 1.06±0.75 | | -1.82±0.76 |
| FRC, 2V_T_ (B) | 4.40±1.78 | 3.43±0.96 | 0.98±1.24 | | -1.68±1.16 |
| FRC, 3V_T_ (C) | 3.73±1.24 * | 3.24±0.97 * | 0.49±0.57 | | -1.08±0.52 * |
| ANOVA | p =0.006  * p =0.008 vs. A, p=0.02 vs. B | p =0.02  * p=0.02 vs. A | p =0.04 | | p =0.002  * p =0.002 vs. A; p =0.007 vs. B |
| Mid iso-volume | | | | | |
| FRC, 2V_T_ (B) | 3.42±1.37 | 2.94±0.85 | 0.48±0.71 | -1.53±0.75 | |
| FRC, 3V_T_ (C) | 3.17±1.09 | 2.83±0.87 | 0.34±0.37 | -1.22±0.46 ^╫^ | |
| FRC_+1VT_, V_T_ (D) | 3.43±1.09 | 3.02±0.87 | 0.41±0.52 | -1.32±0.62 | |
| FRC_+1VT_, 2V_T_ (E) | 3.37±1.06 | 3.00±0.90 | 0.37±0.55 | -1.11±0.57 | |
| ANOVA | p =0.27 | p =0.19 | p =0.60 | p =0.002  ^╫^ p=0.02 vs. B; * p=0.002 vs. B | |
| High iso-volume | | | | | |
| FRC, 3V_T_ (C) | 2.58±0.98 | 2.48±0.80 | 0.10±0.36 | -1.37±0.40 | |
| FRC_+1VT_, 2V_T_ (E) | 2.64±0.77 | 2.52±0.68 | 0.12±0.29 | -1.26±0.41 | |
| FRC_+2VT_, V_T_ (F) | 2.62±0.92 | 2.56±0.75 | 0.06±0.35 | -1.18±0.34 | |
| ANOVA | p =0.93 | p =0.86 | p =0.70 | p =0.07 | |

V_T_, tidal volume; BF, breathing frequency; _E_, minute ventilation; FRC, functional residual capacity. Other abbreviations as in table 1. Data are mean ± SD.
